# Rate of psychiatric disorders and associations with quality of life among community members following the Kaohsiung gas explosion: an 18-month cross-sectional follow-up study

**DOI:** 10.1186/s12955-018-1076-7

**Published:** 2019-01-11

**Authors:** Vincent Shieh, Joh-Jong Huang, Tsyr-En Grace Wu, Ju-Yu Chiu, Yi-Chen Chen, Guijing Lin, Chao-Yueh Su, Frank Huang-Chih Chou

**Affiliations:** 1 Graduate Institute of Gender Education, National Kaohsiung Normal University, Kaohsiung City, Taiwan; 2 Kaohsiung City Government Department of Health, Kaohsiung City, Taiwan; 3 Department of Theology, Chang Jung Christian University, Tainan City, Taiwan; 4 Department of Surgery, Tainan Municipal AN-NAN Hospital-China Medical University, Tainan City, Taiwan; 5 Department of Nursing, Meiho University, Ping-Tong County, Taiwan; 6 Department of Nursing, Kaohsiung Municipal Kai-Syuan Psychiatric Hospital, Kaohsiung City, Taiwan; 7 Department of Nursing, I-Shou University, Kaohsiung County, Taiwan; 8 Superintendent office, Kaohsiung Municipal Kai-Syuan Psychiatric Hospital, 130 Kai-Syuan 2nd Rd, Lingya District, Kaohsiung City, Taiwan

**Keywords:** Depressive disorder, Stress disorders, Post-traumatic, Quality of life

## Abstract

**Objective:**

To conduct a follow-up on the rate and related risk factors of probable disaster-related psychiatric disorders such as depressive disorder (major depressive episode, MDE), stress disorders, post-traumatic (posttraumatic stress disorder, PTSD), and the quality of life of the survivors of a fossil gas explosion in Taiwan 18 months after the event.

**Methods:**

A community-based survey of residents of a community that experienced a petrochemical gas explosion with cross-sectional assessments was conducted 18 months after the event. Two screening tools, including the Disaster-Related Psychological Screening Test (DRPST) and Short Form 12v2 (SF-12v2), were used to survey a representative sample of 388 participants.

**Results:**

The average age of 388 participants is 43.27 ± 15.98 years (males: 203, average age: 41.44 ± 15.74 years; females: 185; average age: 45.27 ± 16.03 years). Probable PTSD, probable MDE, probable PTSD and MDE, and non-PTSD or non-MDE (non-P or -M) were present in 34 (8.8%), 14 (3.6%), 9 (2.3%), and 331 (85.3%) participants, respectively. The significant associated factor for probable PTSD or MDE among those who experienced disaster was financial problems. The associated factors on different quality of life subscales were old age, physical injury, employment, educational level, financial problems, probable PTSD and probable MDE.

**Conclusion:**

While participants’ psychiatric status improved after 18 months, their quality of life continued to be affected, especially the quality of life of those with probable PTSD combined with MDE. Postdisaster treatment and follow-up should be addressed to a greater degree, especially for victims with mental illness, physical injuries and financial problems.

## Introduction

A major petrochemical gas explosion occurred in the Lingya District, Kaohsiung City, Taiwan. That event is known as the “81 petrochemical gas explosion of August 1, 2014.” [1] The incident caused traffic disruptions, casualties, and environmental damage [1, 2]. Three hundred seventy-two casualties were acknowledged as of October 6, 2014, including 32 deaths and 340 serious injuries [1]. This highly stressful event had a severe impact on victims, especially in terms of their mental health.

Many studies [3–7] have shown evidence of psychiatric impairment in disaster survivors. The most common disaster-related psychiatric diagnoses are major depressive episode(MDE) and posttraumatic stress disorder(PTSD), which are strongly associated [3, 8]—an issue that continues to gain attention in trauma outcome research. [3] The prevalence of psychiatric diseases, especially PTSD and MDE, is highest at the beginning of a disaster and gradually declines after mental rehabilitation. In addition, rescue workers such as nurses, fire fighters, and soldiers incur a high prevalence of psychiatric disorders after disaster rescue.

Physical and psychological trauma after various disasters has been shown to significantly impact the quality of life of survivors of various disasters, and physical damage is strongly related to psychological trauma [1, 2, 9–16]. However, three symptoms of PTSD—namely, re-experiencing, avoidance and numbing, and hyperarousal—are common and may be dismissed as unimportant behavioral shifts, or some physiologic reactivity at exposure to cues that symbolize trauma was under-reported [15, 17, 18]. For example, when a person hears the sound of firecrackers, he or she might be too scared to go out the door. Another might have “hypervigilance”, which is a symptom of hyperarousal syndromes. Although the prevalence of PTSD tends to decrease over time among burn survivors, rates of PTSD have been found to increase over time from injuries due to delayed onset or repercussions from the traumatic event (i.e., job loss, health problems) [15, 19]. Therefore, regardless of the mental affliction involved, early detection and longer periods of effective treatment are most important after a disaster.

We know that physical and mental health are interrelated [10] and that physical injuries and material losses are also strongly associated with PTSD [13, 14]. Long-term financial problems are one of the many important factors affecting quality of life (QoL) [8, 12, 20, 21]. Health-related quality of life (HRQoL) includes both physical and mental dimensions [22]. A composite measure of physical and psychological health is HRQoL [19], as assessed by short form-12 or short form-36. Following a disaster, psychological problems have a greater impact on quality of life than injuries do [23]. We know that physical and mental healthcare are interrelated [10] and that physical injuries and material losses are also strongly associated with PTSD [13, 14]. These two dimensions are included in health-related quality of life (HRQoL) [22]. A composite measure of physical and psychological health is HRQoL, as assessed, for example, by short form-12 or − 36 [23]. Experiencing long-term financial problems is one of several important factors affecting quality of life [10, 12, 20, 21]. Through this continuous cycle, trauma may become a personal problem that evolves into a family problem. Therefore, early detection and assistance can reduce the incidence of such problems and improve the QoL of participants and individuals. Furthermore, long-term follow-up is necessary for mental rehabilitation. In the face of a catastrophic disaster, many people may experience psychiatric impairment and poorer quality of life, making it important to give them support, such as mental health rehabilitation, to decrease later sequalae. Mental health rehabilitation involves provision of both economic and health information, lifestyle modification, exercise, emotional support, psychiatric services (including, inter alia, drug treatment and psychotherapy), and stress/adversity management, among other factors. The health organization and study team carried out disaster mental health rehabilitation after the 81 petrochemical gas explosion and simultaneously established a disaster psychological assistance group with the support of the Kaohsiung City government. We performed “walk around” care (a mental health term that denotes active community outreach instead of traditional OPD to offer psychiatric services and mental health information) to provide the victims of the event with emergency mental health services.

A support system is known to be particularly important after a disaster [13]. Accordingly, we conducted a series of studies to investigate the rate of and associated factors for probable disaster-related psychiatric disorders such as MDE, PTSD, and quality of life (QoL) after six [1] and 12 months [2]. In total, 7.4–13.9% (6 months) [1] and 2.9–10.3% (12 months) [2] of individuals with probable PTSD and/or probable MDE showed a reduced quality of life. The reconstruction of one’s life after a disaster can be a challenging process. Mental rehabilitation is a part of this reconstruction process and requires a planned and comprehensive approach [16]. Our research hypotheses are: (1) disasters would increase psychiatric impairment and decrease quality of life in disaster survivors, highlighting the importance of mental rehabilitation, which consists of both short-term intervention and long-term follow-up and referral of cases to regular psychiatric treatment; and (2) that individuals would also benefit from mental rehabilitation [24, 25]. After a disaster, PTSD can have severe and painful effects and must be addressed with appropriate treatment and assistance to enable rapid screening so that victims can receive an early diagnosis and long-term complications can be prevented. Persistent follow-up is necessary to understand the status of the affected after a disaster and to strengthen their support systems. Subsequently, it is important to continue to track the needs of those affected after a disaster to help provide relevant information so that traumatized victims can return to society as quickly as possible. The purpose of this study was to investigate the prevalence of psychiatric disorders (PTSD and MDEs) and associations with QoL in individuals who were exposed to the petrochemical gas explosion, either directly or indirectly.

## Method

### Participants

In this study, the authors selected community residents and subjects affected by a fossil gas explosion within 18 months after the event as research participants. The Kaohsiung municipal government health bureau created a petrochemical gas explosion public information file, which focused on the disaster after the end of the acute period. The participants (including affected individuals, injured individuals and disaster relief personnel) completed the Disaster-Related Psychological Screening Test (DRPST) and SF-12v2 with the help of research assistants, specifically, semiprofessional care providers who completed a standard 2-week training course. The psychiatric training program included the following: 1) how to use questionnaires to collect data; 2) ways to introduce the purpose of the study to the residents and establish rapport; 3) an introduction to psychiatric disorders; and 4) demonstrations of the interview technique by psychiatrists.

The inclusion criteria at the beginning of the 6-month postdisaster survey included the following: 1) people who lived near the disaster site when the gas explosion occurred; 2) those who were injured as they passed the site where the explosion took place; and 3) rescue workers who were in the vicinity before the explosion and were injured after the gas explosion. The exclusion criteria included the following: 1) for those who experienced the petrochemical gas explosion, the occurrence of insomnia, the nature of medical treatment, and a diagnosis of psychiatric disorder by specialists in the previous year; 2) unrelated mental disorders and previous exposure to other disasters; and 3) not living in the study community and not witnessing the disaster.

### Procedure

This was a follow-up study with a cross-sectional survey, and the surveyed participants were affected by this event. Following the occurrence of the gas explosion, we offered psychiatric services for more than 2 years and surveyed these residents at six [1], 12 [2] and 18 months. Because it is difficult to survey a sufficient number of people more than twice during the postdisaster period, we used a dynamic population (persons who lived in the same community and who may or may not have been surveyed in the first survey) instead of surveying the same individuals on two or more occasions [2]. The community residents expressed some resistance to repeat surveys or refused to complete the survey to avoid psychiatric stigma, especially 18 months postdisaster. The number of participants gradually decreased in the series of follow-up studies. However, the number of participants surveyed at 18 months was less than the number surveyed during the first half of the year (388 vs 502). The study was approved by the Institutional Review Board of Kaohsiung Municipal Kai-Syuan Psychiatric Hospital (KSPH-2015-05-R2), and all participants provided informed consent.

### Instruments

In this study, we used the DRPST developed by Chou et al. [26], which is a rapid screening scale for MDEs and PTSD supplemented with information regarding background and associated factors. The DRPST was initially designed to enable the effective and rapid screening of MDE and PTSD among disaster survivors [11, 18, 27] and policemen/firefighters [1, 25, 28] (including 17 items for PTSD and 9 items for MDEs) according to the DSM-IV criteria. A seven-symptom scale (1. distressing dreams of the trauma; 2. acting or feeling as if the trauma were recurring (“flashbacks”); 3. physical distress at exposure to cues that symbolize the trauma; 4. efforts to avoid activities, places, people that arouse recollections of the trauma; 5. efforts to avoid thoughts, feelings associated with the trauma; 6. diminished interest in activities; and 7. hypervigilance) and a three-symptom analog scale (1. depressed mood most of the day, nearly every day; 2. fatigue or loss of energy nearly every day; and 3. feelings of worthlessness or excessive or inappropriate guilt nearly every day) were selected for PTSD and MDE screening, respectively. Scores of 4 or higher on the PTSD scale were used to define the group of participants with PTSD; a score of 2 or higher on the MDE scale was used to define cases with MDEs. This method has been validated in previous studies [11, 18, 25–28]. The SF-12v2 is one of the most commonly used HRQoL questionnaires, and it has become widely used in community-based health surveys and physical and mental illness outcome assessments due to its brevity and psychometric performance [29, 30]. The mental component summary (MCS) and physical component summary (PCS) exhibited high reliability (Cronbach’s α > 0.80) [1]. The summaries estimated health-related functions on the following eight subscales: physical functioning (PF), role limitations caused by physical problems (RP), bodily pain (BP), general health (GH), role limitations caused by emotional problems (RE), vitality (VT), social functioning (SF), and mental health (MH). All 8 scales were used to score PCS and MCS, which were calculated by multiplying by different coefficients. A high score is indicative of high QoL [31, 32].

### Data analysis

The DRPST classification of four groups was as follows: no probable PTSD or MDE was defined as non-P or non-M; an answer of yes to any four questions concerning PTSD on the DRPST was defined as probable PTSD; and an answer of yes to any two questions concerning MDE on the DRPST was defined as probable MDE. Participants whose answers categorized them as both probable PTSD and probable MDE were defined as probable PTSD and MDE. After summing Likert-type scale items on the SF-12v2 survey, each scale was standardized (with scores from 0 to 100, reflecting the lowest and highest levels of functioning, respectively). All data were analyzed using statistical software (SPSS version 17.0). A chi-square test and one-way analysis of variance (ANOVA) with Scheffé’s method for postexamination were used to analyze the victims’ demographic data (such as sex, age, physical injuries, occupation, current marital status, education level, religious belief, and financial problems) and differences between QoL and the four respondent groups. In addition, we used logistic regression analysis to examine the demographic data of the victims, probable psychiatric impairment, and gas explosion-associated factors; multiple regression analysis was used to examine relationships between the victims’ demographic data, possible PTSD, possible MDE and QoL (PF, RP, BP, GH, VT, SF, RE, MH, PCS and MCS).

## Results

After 18 months, only community dwellers were included. Ultimately, 388 participants were included (average age: 43.27 ± 15.98 years; males: 203, average age: 41.44 ± 15.74 years; females: 185; average age: 45.27 ± 16.03 years). Overall, 331 of the 388 respondents (85.3%) were assigned to the non-P or non-M group. For a comparison of the rates of PTSD and MDE at the three time points, we also used demographic data obtained in two previous studies as a representative sample of 502 participants (average age: 42.90 ± 16.61 years; *M*: 270, average age: 40.89 ± 16.40 years; *F*: 232; average age: 45.25 ± 16.58 years) 6 months after the event and a representative sample of 486 participants (average age: 42.89 ± 16.05 years; M: 255, average age: 40.68 ± 15.92 years; F: 231; average age: 45.32 ± 16.20 years) 12 months after the event (please see others in Table 1).

**Table 1:** Demographic data on respondents with probable psychiatric diseases among postgas explosion respondents in three study periods

	Non-PTSD or MDE		Probable PTSD		Probable MDE		Probable PTSD and MDE		χ^2^ or *t*-test
	*n*	rate (%)	*n*	rate (%)	*n*	rate (%)	*n*	rate (%)	
Sex^a^									
6 months (502)									0.063
Male	195	72.2	24	8.9	14	5.2	37	13.7	
Female	146	62.9	30	12.9	23	9.9	33	14.2	
12 months (486)									0.081
Male	214	83.9	22	8.6	4	1.6	15	5.9	
Female	174	75.3	28	12.1	10	4.3	19	8.2	
18 months (388)									0.383
Male	178	87.7	13	6.4	7	3.4	5	2.5	
Female	153	82.7	21	11.4	7	3.8	4	2.2	
Age^b^ (Mean ± SD)									
6 months	42.1	16.8	47.0	17.0	41.9	17.9	44.0	14.6	0.221
12 months	42.4	16.5	44.6	17.5	45.0	15.8	45.7	9.7	0.529
18 months	42.9	16.0	47.0	16.3	45.4	16.9	40.9	8.7	0.477
Current marital status^a^									
6 months (502)									0.445
Single	161	66.3	24	11.0	18	8.4	40	14.3	
Married/Living together	180	69.5	30	10.6	19	6.9	30	13.8	
12 months (486)									0.006^*^
Single	184	80.7	29	12.7	8	3.5	7	3.1	
Married/Living together	204	79.1	21	8.1	6	2.3	27	10.5	
18 months (388)									0.728
Single	152	83.5	19	10.4	7	3.8	4	2.2	
Married/Living together	179	86.9	15	7.3	7	3.4	5	2.4	
Financial problems^a^									
6 months (502)									< 0.001^*^
Yes	246	76.2	28	8.7	23	7.1	26	8.0	
No	95	53.1	26	14.5	14	7.8	44	24.6	
12 months (486)									< 0.001^*^
Yes	314	85.6	25	6.8	11	3.0	17	4.6	
No	74	62.2	25	21.0	3	2.5	17	14.3	
18 months (388)									< 0.001^*^
Yes	257	92.4	8	2.9	9	3.2	4	1.4	
No	74	67.3	26	23.6	5	4.5	5	4.5	

Notes: ^a^ Chi-square test; ^b^ One-way ANOVA; ^*^ *p* < 0.05

Fifty-seven of the 388 respondents (14.7%) had at least one probable psychiatric diagnosis and were assigned to the following groups: probable PTSD (*n* = 34) [8.8%], probable MDE (*n* = 14) [3.6%], and probable PTSD and MDE (*n* = 9) [2.3%]. We found that respondents who survived the postgas explosion differed significantly from those who did not experience the event in terms of financial problems. Additionally, financial problems were significantly more frequent in the probable PTSD and MDE group than in the non-P or non-M group (*p* < 0.001) (Table 2).

**Table 2:** Demographic data on participants with psychiatric diseases

	Non-PTSD or MDE		Probable PTSD		Probable MDE		Probable PTSD & MDE		*p*
	*n* = 331	Rate(%)	*n* = 34	Rate(%)	*n* = 14	Rate(%)	*n* = 9	Rate(%)	
Sex^a^									0.383
Male	178	87.7	13	6.4	7	3.4	5	2.5	
Female	153	82.7	21	11.4	7	3.8	4	2.2	
Age^b^									0.477
(Mean ± SD)	42.9	16.0	47.0	16.3	45.4	16.9	40.9	8.7	
Physical Injuries^a^									0.680
No	204	86.4	21	8.9	8	3.4	3	1.3	
Mild or Moderate	79	84.9	8	8.6	3	3.2	3	3.2	
Severe	48	81.4	5	8.5	3	5.1	3	5.1	
Working status									0.801
Unemployed	107	84.3	13	10.2	5	3.9	2	1.6	
Employed	224	85.8	21	8.0	9	3.4	7	2.7	
Current Marital Status									0.728
Single	152	83.5	19	10.4	7	3.8	4	2.2	
Married/Living Together	179	86.9	15	7.3	7	3.4	5	2.4	
Educational Level									0.051
Junior High School or Below	66	78.6	14	16.7	3	3.6	1	1.2	
Senior High School	119	88.8	9	6.7	5	3.7	1	0.7	
College or Higher	146	85.9	11	6.5	6	3.5	7	4.1	
Religious Beliefs									0.755
No	102	84.3	13	10.7	4	3.3	2	1.7	
Yes	229	85.8	21	7.9	10	3.7	7	2.6	
Financial Problems									< 0.001^*^
No	257	92.4	8	2.9	9	3.2	4	1.4	
Yes	74	67.3	26	23.6	5	4.5	5	4.5	

Note: ^a^Chi-square test; ^b^One-way ANOVA; ^*^p < 0.001

The only factor found to be significantly associated with probable psychiatric disorder in respondents was financial problems, which showed a significant positive association with probable psychiatric disorders. The association of probable psychiatric disorders was greater in those with financial problems (OR = 6.344; *p* < 0.001) than in those without financial problems (Table 3).

**Table 3:** Associated factors in postgas explosion among respondents with psychiatric impairment

	Odds ratio	95% CI		*p*
Sex				
Female	ref.			
Male	1.470	0.754	2.864	0.258
Age				
(Mean ± SD)	1.021	0.995	1.048	0.110
Physical Injuries				
No	ref.			
Mild or Moderate	1.487	0.686	3.224	0.314
Severe	1.534	0.654	3.602	0.325
Employment				
No	ref.			
Yes	0.949	0.470	1.919	0.885
Current Marital Status				
Single	ref.			
Married/Living Together	0.732	0.366	1.463	0.377
Educational Level				
Junior High School or Below	ref.			
Senior High School	0.532	0.224	1.260	0.151
College or Higher	1.085	0.428	2.754	0.863
Religious Beliefs				
No	ref.			
Yes	0.736	0.366	1.484	0.392
Financial Problems				
No	ref.			
Yes	6.344	3.395	11.855	< 0.001^*^

^*^p < 0.001

The QoL scores for the RP, BP, VT, RE, MH and MCS in the non-P or non-M group were significantly higher than the scores of the different probable psychiatric impairment groups (*p* < 0.001). In addition, the QoL scores for the PF, RP, GH and SF in the non-P or non-M group were significantly higher than the scores of the probable PTSD and the probable PTSD and MDE groups (*p* < 0.001). Furthermore, the QoL scores for the PCS in the non-P or non-M group were significantly higher than those in the probable PTSD group (*p* < 0.001). Finally, the QoL scores for the PF, RE, MH and MCS in the probable MDE group were significantly higher than the scores of the probable PTSD and MDE group (*p* < 0.001) (Table 4).

**Table 4:** Comparison of quality of life between each of the groups among the postgas explosion respondents

	Non-PTSD or MDE		Probable PTSD		Probable MDE		Probable PTSD & MDE		*p*	Scheffé’s method
	Mean	SD	Mean	SD	Mean	SD	Mean	SD		
Physical Functioning	90.6	22.1	60.3	33.8	82.1	22.8	66.7	41.5	< 0.001	a > b,d c > d
Role Physical	76.7	24.5	49.3	24.8	64.3	31.3	51.4	34.5	< 0.001	a > b,d
Bodily Pain	89.7	18.5	68.4	28.4	73.2	18.3	58.3	35.4	< 0.001	a > b,c,d
General Health	53.9	26.0	23.5	21.1	35.0	27.0	13.9	13.2	< 0.001	a > b,d
Vitality	69.8	17.5	42.6	18.0	46.4	19.3	27.8	8.3	< 0.001	a > b,c,d
Social Functioning	83.3	17.3	64.7	23.1	69.6	20.0	55.6	17.3	< 0.001	a > b,d
Role Emotional	81.0	18.9	48.5	22.8	65.2	26.9	33.3	30.6	< 0.001	a > b,c,d c > d
Mental Health	77.6	15.1	44.5	18.0	58.0	10.5	37.5	23.4	< 0.001	a > b,c,d c > d
Physical Component Summary	49.7	8.1	41.9	10.9	46.0	7.5	42.7	14.0	< 0.001	a > b
Mental Component Summary	51.2	7.4	37.2	8.4	41.9	7.0	30.1	13.2	< 0.001	a > b,c,d c > d

As shown in Table 5, most subscales were worse for older victims than for younger ones. In addition, QoL scores on the PF (*p* = 0.003), RP (*p* = 0.028), GH (*p* = 0.001) and PCS (*p* < 0.001) subscales among victims with mild or moderate physical injuries were worse than those among victims with no physical injuries. Additionally, most subscales among victims with severe physical injuries were worse than the scores among those with no physical injuries. However, QoL scores on the RE (*p* = 0.018) subscales among victims who had an occupation were better than those among victims who had no occupation. Moreover, QoL scores on the PF (*p* = 0.022), BP (*p* = 0.006) and PCS (*p* = 0.019) subscales among victims with a college or higher educational level were worse than those among victims with a junior high school or below educational level, although QoL scores on the GH (*p* = 0.008) and PCS (*p* = 0.023) subscales were better than those among victims with a junior high school educational level. QoL scores were worse among victims with financial problems on the most subscales. Additionally, QoL scores (all *p* < 0.001) at the PF, RP, BP, GH, VT, SF, RE, MH, PCS and MCS subscales were worse among victims with probable MDE. Finally, among victims with probable PTSD, QoL scores were worse on the most subscales.

**Table 5:** Multiple regression for the prediction of SF-12v2 subscale scores for respondents 12 months postgas explosion (*n* = 388)

	Physical Functioning	Role Physical	Bodily Pain	General Health	Vitality	Social Functioning	Role Emotional	Mental Health	Physical Component Summary	Mental Component Summary
β	106.72^*^	91.33^*^	100.88^*^	70.52^*^	78.85^*^	81.97^*^	86.75^*^	75.75^*^	56.67^*^	50.25^*^
Sex										
Female	ref.	ref.	ref.	ref.	ref.	ref.	ref.	ref.	ref.	ref.
Male	− 4.00	−0.64	0.44	3.84	0.56	0.92	−0.85	0.56	−0.15	0.42
Age	−0.40^*^	−0.36^*^	− 0.27^*^	−0.45^*^	− 0.15^*^	− 0.11	−0.22^*^	− 0.06	−0.16^*^	− 0.02
Physical Injuries										
No	ref.	ref.	ref.	ref.	ref.	ref.	ref.	ref.	ref.	ref.
Mild or Moderate	−8.30^*^	−6.56^*^	−4.33	−10.30^*^	−3.68	2.41	−1.39	−0.14	−3.75^*^	0.83
Severe	−25.42^*^	−24.84^*^	− 19.23^*^	−13.96^*^	−5.70^*^	−5.57^*^	− 8.03^*^	0.01	− 10.27^*^	1.29
Employment										
No	ref.	ref.	ref.	ref.	ref.	ref.	ref.	ref.	ref.	ref.
Yes	4.48	4.72	1.35	1.94	1.43	0.42	5.46^*^	3.02	0.92	1.30
Current Marital Status										
Single	ref.	ref.	ref.	ref.	ref.	ref.	ref.	ref.	ref.	ref.
Married/Living Together	4.08	2.92	2.80	0.03	−0.58	2.76	2.60	1.78	0.89	0.61
Educational Level										
Junior High School or Below	ref.	ref.	ref.	ref.	ref.	ref.	ref.	ref.	ref.	ref.
Senior High School	7.35^*^	5.94	7.53^*^	3.19	2.55	3.93	3.13	4.12	2.60^*^	1.03
College or Higher	6.24	3.59	5.16	9.90^*^	−0.56	3.97	1.93	3.56	2.71^*^	0.61
Religious Beliefs										
No	ref.	ref.	ref.	ref.	ref.	ref.	ref.	ref.	ref.	ref.
Yes	−2.85	−2.10	−2.69	−0.62	−1.57	1.91	0.02	−0.42	−1.03	0.38
Financial problems										
No	ref.	ref.	ref.	ref.	ref.	ref.	ref.	ref.	ref.	ref.
Yes	−0.04	−4.53	−7.09^*^	−6.37^*^	−6.96^*^	−2.32	−6.36^*^	−7.63^*^	−0.88	−3.42^*^
Probable MDE (No/Yes)	−25.38^*^	−20.36^*^	−14.41^*^	− 22.81^*^	−21.61^*^	− 15.32^*^	− 27.79^*^	−26.40^*^	−5.61^*^	− 11.70^*^
Probable PTSD (No/Yes)	− 0.96	−4.83	−12.36^*^	− 15.52^*^	−19.42^*^	− 12.14^*^	−15.03^*^	− 15.54^*^	−1.26	−8.85^*^
Adjusted R^2^	0.31	0.25	0.29	0.29	0.29	0.14	0.30	0.38	0.32	0.33

* < .05

## Discussion

After the gas explosion, the Kaohsiung City government immediately organized a psychiatric team to offer mental health services to residents and victims, including psychiatric services for people with psychiatric impairments (including, inter alia, drug treatment and psychotherapy) and a mental health promotion program that included lifestyle modification and stress/adversity management, among other services, for community residents. Comparing the study outcomes at six [1] and 12 [2] months reveals a slightly decreasing trend in the rate of PTSD. The probable MDE prevalence was 7.4, 2.9, and 3.6%. A decreasing trend of MDE prevalence was observed at 1 year and beyond. The probable prevalence of MDE and PTSD was 13.9, 2.9, and 2.3%. The trend is similar to that of probable MDE. A comparison with previous studies [1, 2] and other disasters [16] in Taiwan showed a high rate of psychiatric diseases within 6–12 months and a slight decrease after 12–36 months. The current research identified that those exposed to the incident reported similar levels of psychological trauma and the presence of associated financial problems, similar to levels reported at 6 and 12 months after the event. [1, 2]. However, there are some associated factors for probable PTSD or MDE, including being female and having experienced a physical injury, at 6 months [1] and 12 months [2]. After a disaster, financial difficulty is highly associated with psychiatric morbidity; in turn, psychiatric morbidity might be the source of financial issues, a finding that is consistent with previous studies on different disasters [1, 2, 11, 13, 21]. Most victims were able to recover from their physical injuries, and their physical functioning was restored. However, the recovery period for psychological trauma is longer than that for physical injuries, causing financial problems and affecting QoL.

When we compared the QoL of each of the different groups among the postgas explosion respondents (Table 3) with that of the respondents after 6 and 12 months in two previous studies [1, 2], we obtained the following results: first, non-P or non-M QoL scores were still higher than those associated with a psychological trauma diagnosis. Second, the PCS scores associated with the non-P or non-M QoL scores were higher than those associated with the probable PTSD scores and probable PTSD and MDE. Third, PF, RE, MH and MCS subscale scores in non-P or non-M individuals were higher than those in probable PTSD and MDE individuals. Among them, PF affected the 6- and 18-month QoL scores. Disasters can cause physical and psychological harm. Extremely stressful events can have a significant effect on mental health, and PTSD and MD are common sequelae [1, 2, 9, 10, 16, 23, 24]. Wu et al. [10] confirmed that physical and mental health in postearthquake victims are highly interrelated. Concurrent psychopathology symptoms, physical illnesses and long-term financial problems are associated with QoL [1, 2, 10, 12, 13]. Thus, we infer that two or more psychiatric diagnoses result in family dysfunction, financial problems, and the emergence of poor QoL scores.

As shown in Table 4, we found that age, physical injuries, financial problems, probable MDE and probable PTSD were significantly negatively correlated with QoL. However, occupation and educational level were significantly positively correlated with QoL. Compared with the results of [1, 2], there were significant negative correlations between MDE and QoL. Next, age had no significant correlation with QoL. Finally, there was no significant correlation between current marital status and religious belief for either sex at any time point. Many studies have found that after a disaster, QoL is negatively correlated with aging, physical illness, financial problems and PTSD/MDE [10, 12, 28]. Furthermore, the persistence of long-term financial problems was one of many important factors affecting QoL [1, 2, 10]. Thus, a lower education level and no occupation will affect disaster victims in terms of financial support to a greater extent than it will affect others [11]. Interestingly, material losses and physical injuries were positively associated with the later development of psychiatric impairments [13]. However, Wu et al. [10] found that older victims’ QoL scores were lower, especially in the physical dimension. Therefore, these findings suggest that aging, severe physical injuries, and a lower educational level in the physical dimension are important negative factors. No employment is correlated with financial problems. Thus, levels of physical injuries and financial problems are correlated with the development of psychiatric impairment. Once again, it is confirmed that regardless of the time postdisaster, physical and psychological factors affect QoL, and early detection of problems and longer provision of assistance are necessary.

Our research hypotheses were that disasters highlighted the importance of mental rehabilitation, which consists of both short-term intervention and long-term follow-up and referral of cases to regular psychiatric treatment, and that affected individuals would benefit from mental rehabilitation. [24, 25] We believe that this study has the following strengths: 1. We provided mental rehabilitation after a major disaster, allowing professionals to offer early intervention and provide continuous follow-up and care or medical treatment. 2. We provided disaster epidemiological data, including associated factors, for public health. However, we found that although the number of psychiatric diseases had decreased compared to that 12 months after the disaster [2], the number of probable MDE cases did not change. Additionally, this study has the following limitations: Those who have experienced a gas explosion may be reluctant to consciously recall the situation at that time without any problems and refuse to be visited. This issue may result in an underestimation of the severity of participants’ psychiatric conditions or an underestimation of the proportion of participants suffering from mental illness. We did not collect information about the participants’ prior mental health histories, which might be a confounder for this study. We also used screening scales instead of employing psychiatrists to survey the participants’ psychiatric disorders, which require more comprehensive mental health assessments that were not able to be undertaken. Because the disaster studied is a man-made disaster (which made it difficult to conduct a well-designed study), the authors acknowledge the potential impact of multicollinearity in the current study, we did not use an adjusted *p* value due to the important clinical implications of the research. Furthermore, we simply used a global “distress” variable instead of PTSD or MDE because of the lack of an adequate number of psychiatrists to provide diagnoses.

## Conclusion

Early intervention was performed in the acute postdisaster phase, resulting in a mild decrease in the number of cases of mental illness 18 months later. Therefore, mental rehabilitation should continue for a longer period. There are still many participants who were profoundly affected by the event, and their QoL was also affected, especially among those with probable PTSD and MDE. However, we also used a limited study design and found a sufficient number of people impacted by the disaster who were willing to participate in our study. In the future, researchers could compare subgroups, focusing on exposure and on those who developed psychiatric morbidity versus those who did not. In addition, we believe that long-term follow-up and regular psychiatric management are needed to help participants successfully reconstruct their lives.

## Abbreviations

BP: Bodily pain
DRPST: Disaster-related psychological screening test
DSM-IV: The Diagnostic and Statistical Manual of Mental Disorders-IV
GH: General health
HRQoL: Health-Related Quality of Life
MCS: Mental component summary
MDE: Major depressive episode
MH: Mental health
PCS: Physical component summary
PF: Physical functioning
PTSD: Posttraumatic stress disorder
QoL: Quality of life
RE: Role Limitations Caused by Emotional Problems
RP: Role Limitations Caused by Physical Problems
SF: Social functioning
SF-12v2: Short form 12v2
VT: Vitality
